# Supplementary material for: CellDART: cell type inference by domain adaptation of single-cell and spatial transcriptomic data
Source: Nucleic Acids Res. 2022 Feb 22;50(10):e57. doi: 10.1093/nar/gkac084 (PMC9177989; doi:10.1093/nar/gkac084)
Supplement: gkac084_Supplemental_Files [file gkac084_supplemental_files.zip › Supplementary Figures_fin.pdf]

Supplementary Figures

CellDART: Cell type inference by domain adaptation of single-cell and spatial transcriptomic data

Sungwoo Bae[1,2,†], Kwon Joong Na[3,4,†], Jaemoon Koh[5], Dong Soo Lee[1,2,6], Hongyoon Choi[2,6,*], Young Tae Kim[3,4,*]

[1]Department of Molecular Medicine and Biopharmaceutical Sciences, Graduate School of Convergence Science and Technology, Seoul National University, Seoul, Republic of Korea.; [2]Department of Nuclear Medicine, Seoul National University Hospital, Seoul, Republic of Korea.; [3]Department of Thoracic and Cardiovascular Surgery, Seoul National University Hospital, Seoul, Republic of Korea; [4]Seoul National University Cancer Research Institute, Seoul National University College of Medicine, Seoul, Republic of Korea; [5]Department of Pathology, Seoul National University Hospital, Seoul, Republic of Korea; [6]Department of Nuclear Medicine, Seoul National University College of Medicine, Republic of Korea

[†]These authors contributed equally to this work

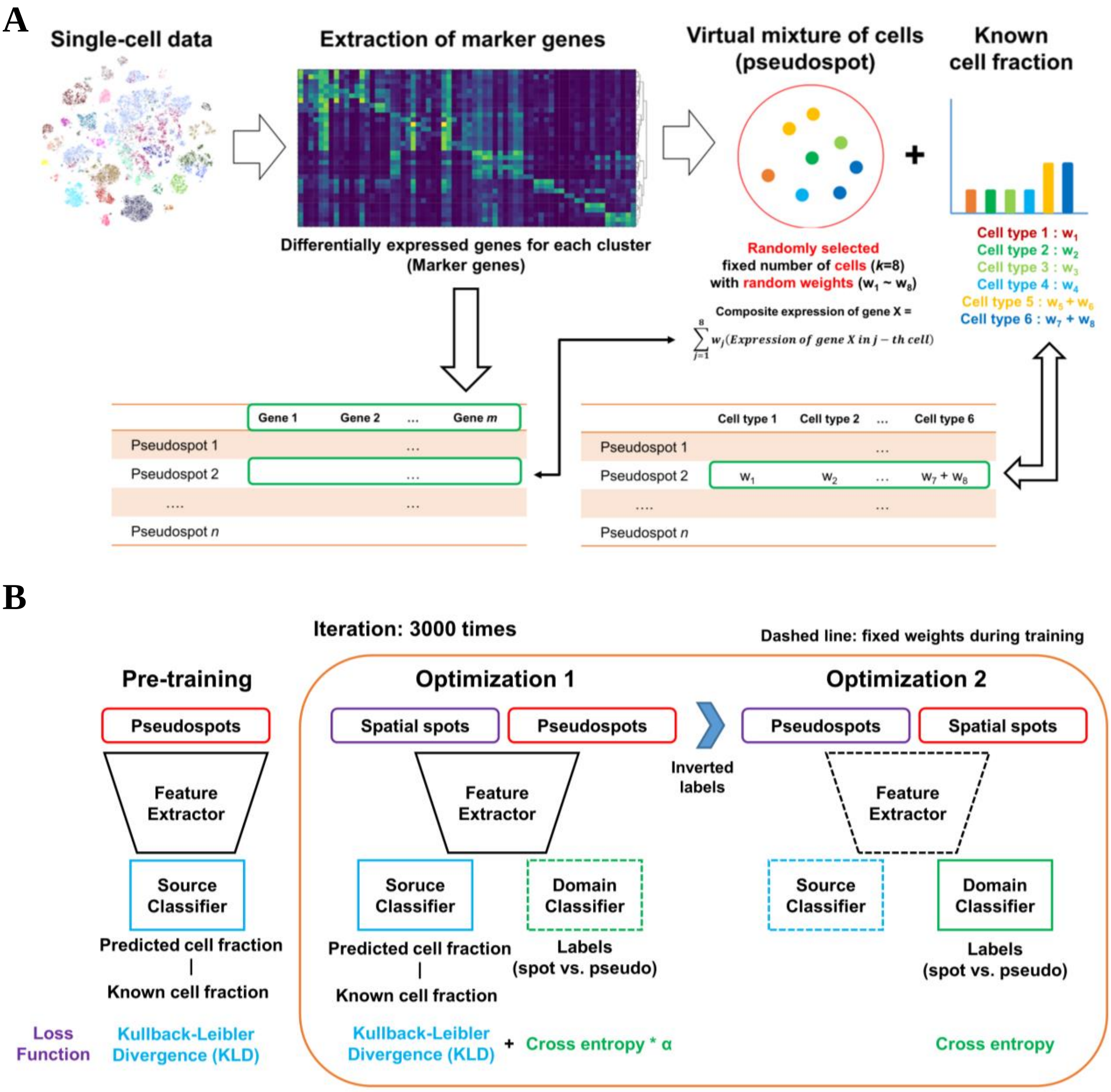

Figure S1: Detailed workflow of CellDART analysis.

(A) The top *l* (*l* = 20 for brain and *l* = 10 for lung) differentially expressed genes for each single-cell cluster were pooled to form a total of *m* marker genes. The fixed number of cells (*k* = 8 for brain Visium, *k* = 2 for brain Slide-seq, and *k* = 10 for lung) were randomly sampled from the single-cell data and random weights (w_1, w_2, ···, w_k) were given to each cell. The consequent mixture of the cells was called a ‘pseudospot’. The weighted sums were calculated for each marker gene expression in cells comprising a pseudospot. Also, the cell fraction was calculated by adding the weights given to each cell-type. The process was repeated to generate a total of

n pseudospots.

(B) The training process was composed of two parts. In the pre-training, a feature extractor (*f*) with a source classifier (*S*) was trained to predict cell fraction. The neural network was trained such that loss function (L_s), Kullback-Leibler divergence (KLD), between predicted and known cell fraction, is minimum. For the first optimization process, the feature extractor (*f*) with the source (*S*) and the domain classifier (*D*) was jointly trained to minimize the total loss (L_{total}) represented as the weighted sum of KLD (L_s) and cross-entropy ($L_{adv,1}$). The weights of the domain classifier were fixed during the optimization. In the second optimization process, only the domain classifier was updated with the inverted label for spots and pseudospots ($L_{adv,2}$). The two optimization processes were iterated 3000 times during the training.

A

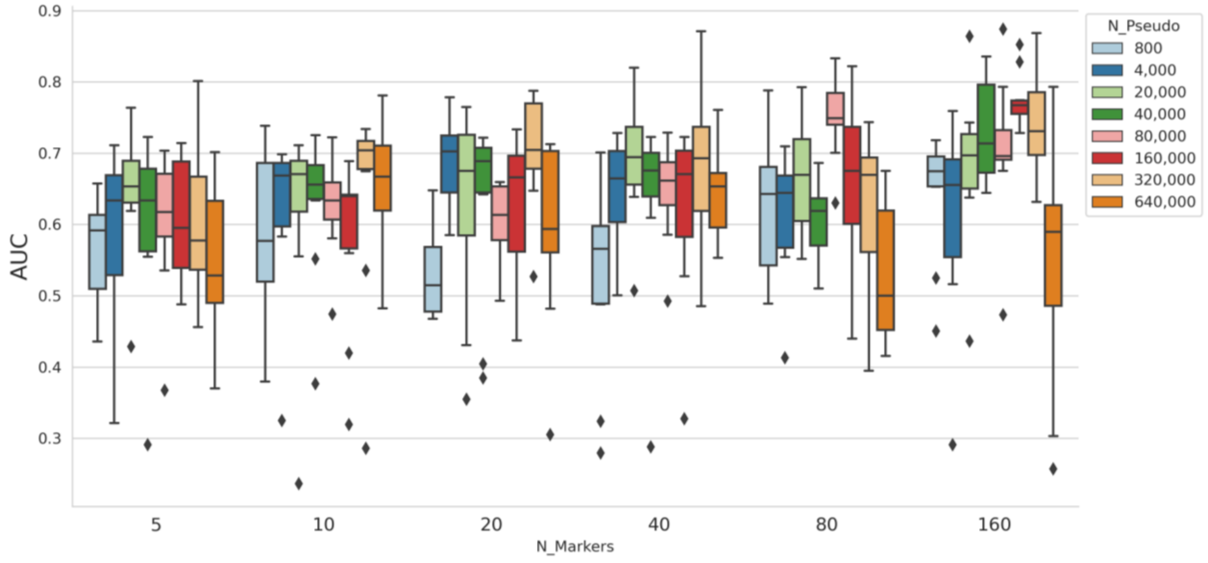

AUC
0.9
0.8
0.7
0.6
0.5
0.4
0.3
5
10
20
40
80
160
N_Markers
N_Pseudo
800
4,000
20,000
40,000
80,000
160,000
320,000
640,000

B

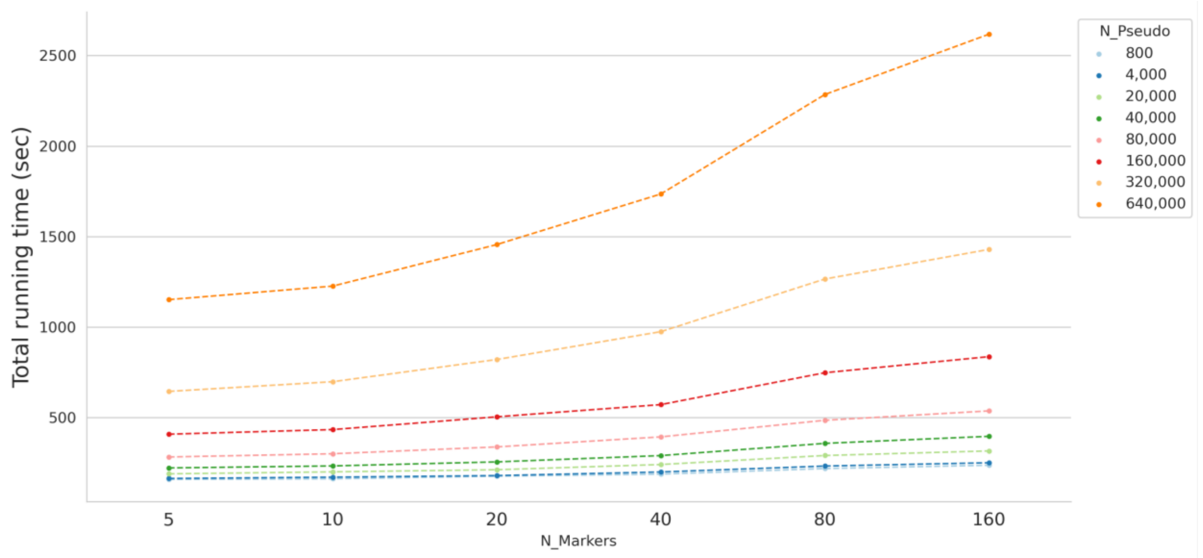

Total running time (sec)
2500
2000
1500
1000
500
5
10
20
40
80
160
N_Markers
N_Pseudo
800
4,000
20,000
40,000
80,000
160,000
320,000
640,000

Figure S2: Evaluation of CellDART performance according to parameters

(A) CellDART models with different parameters (number of marker genes in each cell cluster and number of pseudospots) were tested using prediction accuracy of layer-specific excitatory neuron distribution. Area under the curve (AUC) values were calculated for CellDART models with a different number of marker genes (N_markers) and pseudospots (N_pseudo). The results across the ten excitatory neuron clusters were pooled and visualized in each boxplot.

(B) The total running time for CellDART models with a different number of marker genes (N_markers) and pseudospots (N_pseudo) were visualized with a line plot.

A

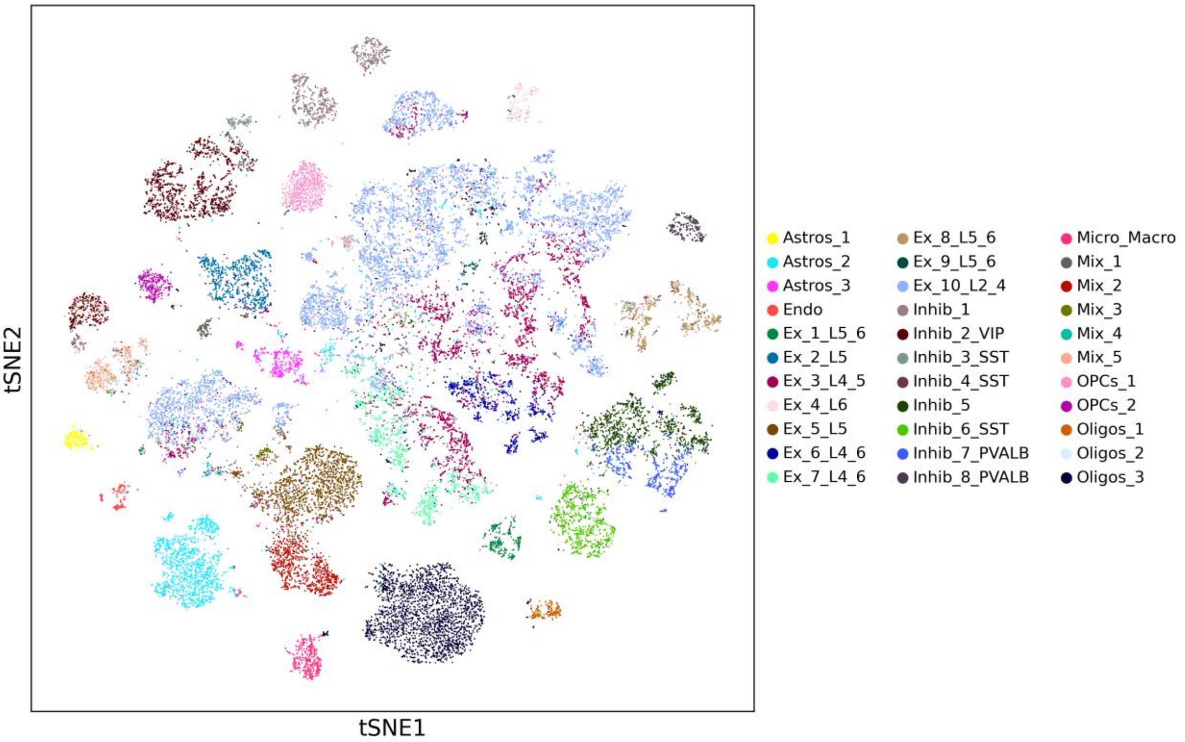
tSNE2
tSNE1
Astros_1
Astros_2
Astros_3
Endo
Ex_1_L5_6
Ex_2_L5
Ex_3_L4_5
Ex_4_L6
Ex_5_L5
Ex_6_L4_6
Ex_7_L4_6
Ex_8_L5_6
Ex_9_L5_6
Ex_10_L2_4
Inhib_1
Inhib_2_VIP
Inhib_3_SST
Inhib_4_SST
Inhib_5
Inhib_6_SST
Inhib_7_PVALB
Inhib_8_PVALB
Micro_Macro
Mix_1
Mix_2
Mix_3
Mix_4
Mix_5
OPCs_1
OPCs_2
Oligos_1
Oligos_2
Oligos_3

B

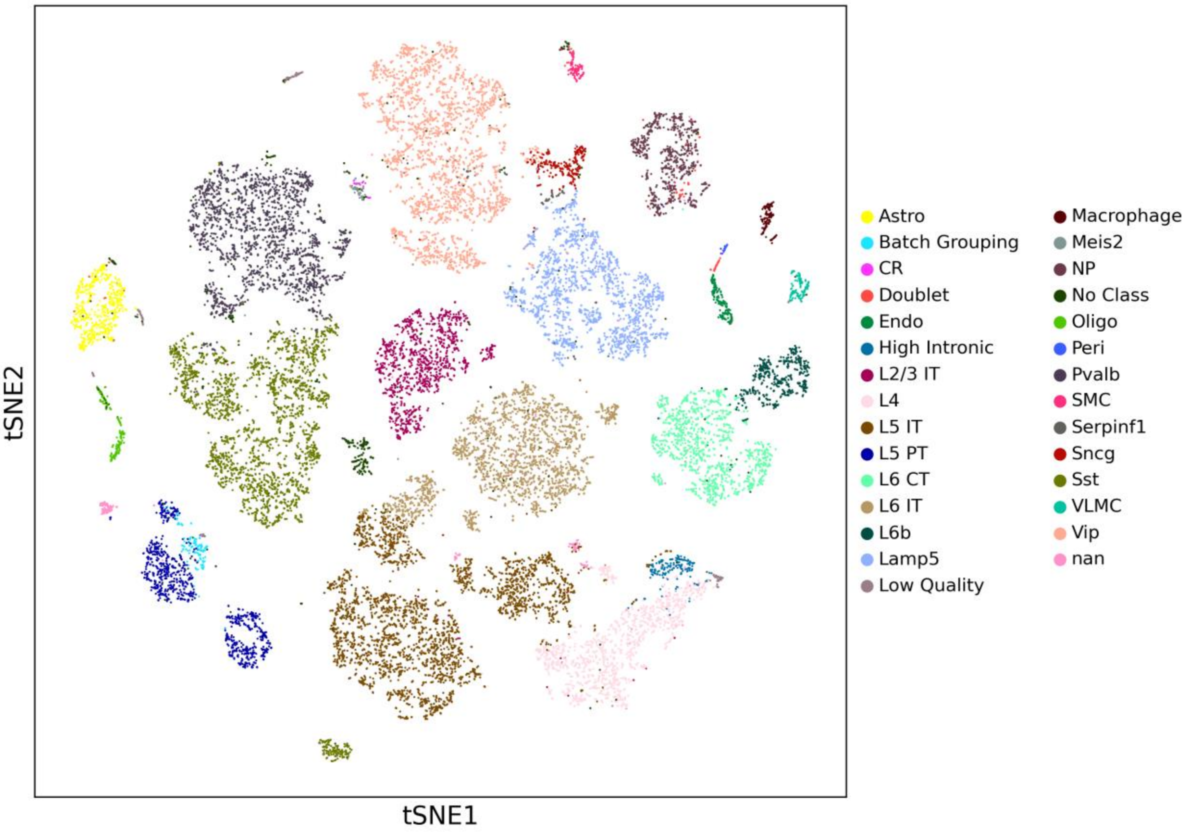
tSNE2
tSNE1
Astro
Batch Grouping
CR
Doublet
Endo
High Intronic
L2/3 IT
L4
L5 IT
L5 PT
L6 CT
L6 IT
L6b
Lamp5
Low Quality
Macrophage
Meis2
NP
No Class
Oligo
Peri
Pvalb
SMC
Serpinf1
Sncg
Sst
VLMC
Vip
nan

C

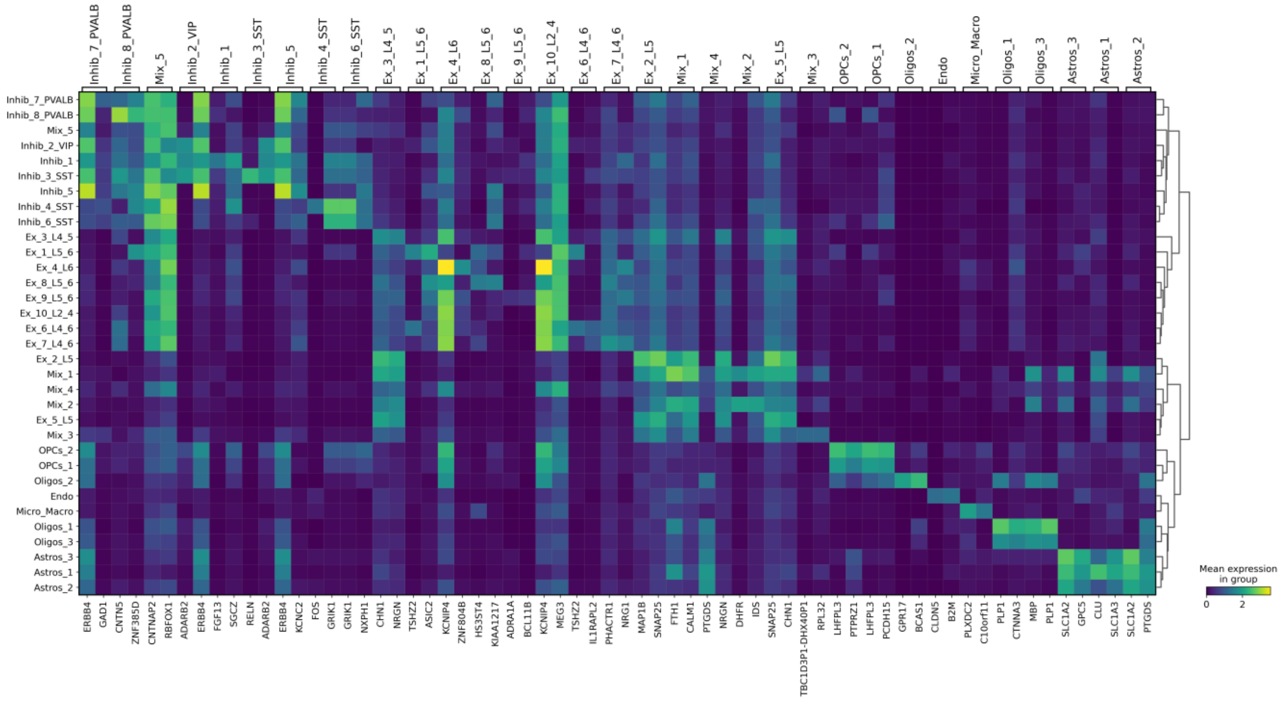

Inhib_7_PVALB
Inhib_8_PVALB
Mix_5
Inhib_2_VIP
Inhib_1
Inhib_3_SST
Inhib_5
Inhib_4_SST
Inhib_6_SST
Ex_3_L4_5
Ex_1_L5_6
Ex_4_L6
Ex_8_L5_6
Ex_9_L5_6
Ex_10_L2_4
Ex_6_L4_6
Ex_7_L4_6
Ex_2_L5
Mix_1
Mix_4
Mix_2
Ex_5_L5
Mix_3
OPCs_2
OPCs_1
Oligos_2
Endo
Micro_Macro
Oligos_1
Oligos_3
Astros_3
Astros_1
Astros_2
ERBB4
GAD1
CNTN5
ZNF385D
CNTNAP2
RBFOX1
ADARB2
ERBB4
FGF13
SGCZ
RELN
ADARB2
ERBB4
KCNC2
FOS
GRIK1
GRIK1
NXPH1
CHN1
NRGN
TSHZ2
ASIC2
KCNIP4
ZNF804B
HS3ST4
KIAA1217
ADRA1A
BCL11B
KCNIP4
MEG3
TSHZ2
IL1RAPL2
PHACTR1
NRG1
MAP1B
SNAP25
FTH1
CALM1
PTGDS
NRGN
DHFR
IDS
SNAP25
CHN1
TBC1D3P1-DHX40P1
RPL32
LHFPL3
PTPRZ1
LHFPL3
PCDH15
GPR17
BCAS1
CLDN5
B2M
PLXDC2
C10orf11
PLP1
CTNNA3
MBP
PLP1
SLC1A2
GPC5
CLU
SLC1A3
SLC1A2
PTGDS
Mean expression in group
0
2

D

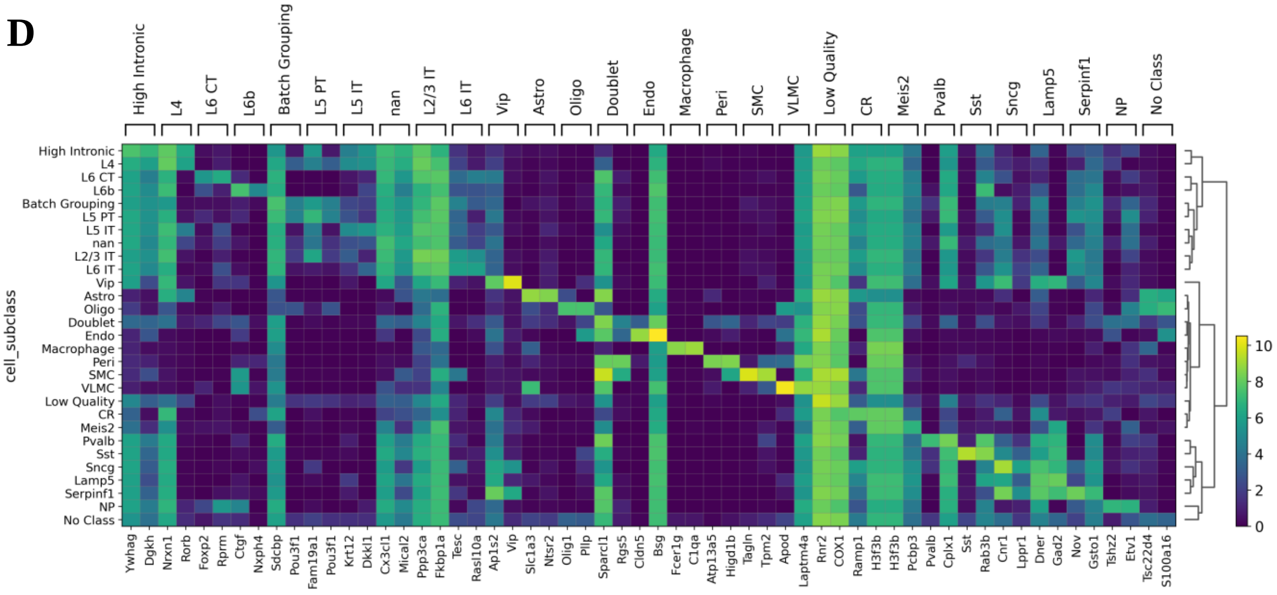

High Intronic
L4
L6 CT
L6b
Batch Grouping
L5 PT
L5 IT
nan
L2/3 IT
L6 IT
Vip
Astro
Oligo
Doublet
Endo
Macrophage
Peri
SMC
VLMC
Low Quality
CR
Meis2
Pvalb
Sst
Sncg
Lamp5
Serpinf1
NP
No Class
cell_subclass
Ywhag
Dgkh
Nrxn1
Rorb
Foxp2
Rprm
Ctgf
Nxph4
Sdcbp
Pou3f1
Fam19a1
Pou3f1
Krt12
Dkkl1
Cx3cl1
Mical2
Ppp3ca
Fkbp1a
Tesc
Rasl10a
Ap1s2
Vip
Slc1a3
Ntsr2
Olig1
Plp
Sparcl1
Rgs5
Cldn5
Bsg
Fcer1g
C1qa
Atp13a5
Higd1b
Tagln
Tpm2
Apod
Laptm4a
Rnr2
COX1
Ramp1
H3f3b
H3f3b
Pcbp3
Pvalb
Cplx1
Sst
Rab3b
Cnr1
Lppr1
Dner
Gad2
Nov
Gsto1
Tshz2
Etv1
Tsc22d4
S100a16
0
2
4
6
8
10

Figure S3: Preprocessing mouse brain and human dorsolateral prefrontal cortex (DLPFC) single-cell data

(A-B) t-SNE map for gene expression in (A) human DLPFC and (B) mouse brain single-cell data. The identity of the 33 and 29 clusters is color-coded and presented on the right side of the plot.

(C-D) Heatmaps for average log-normalized gene expression in the top 2 marker genes of each cell cluster in (C) human DLPFC and (D) mouse brain tissues. Hierarchical clustering was performed for the 33 and 29 cell types based on the expression profiles of the presented genes.

A

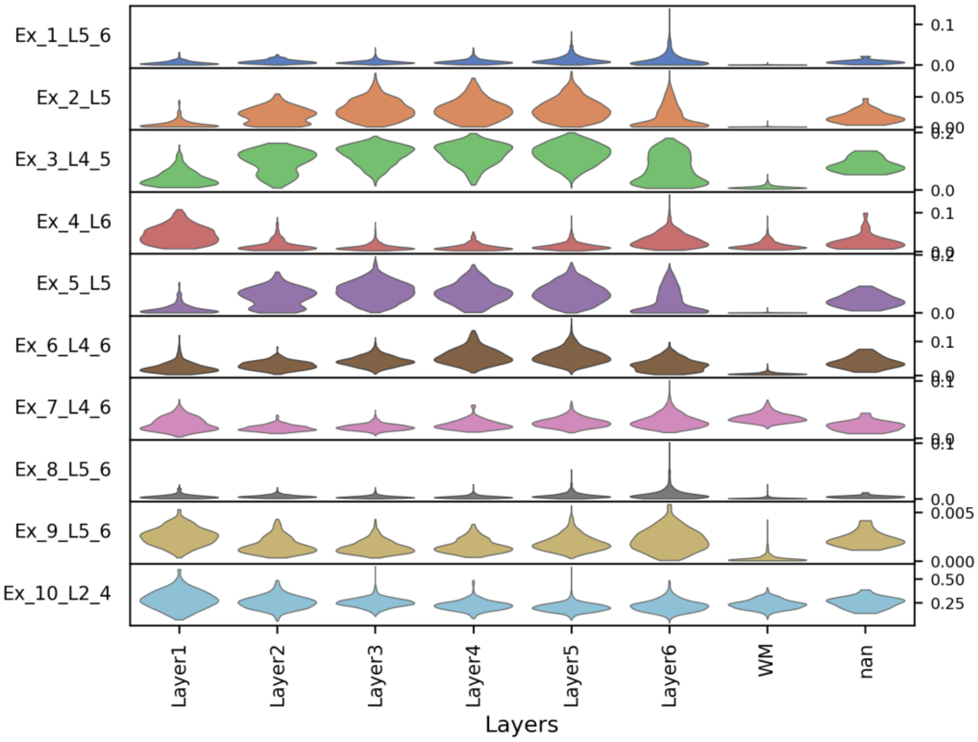

Ex_1_L5_6
Ex_2_L5
Ex_3_L4_5
Ex_4_L6
Ex_5_L5
Ex_6_L4_6
Ex_7_L4_6
Ex_8_L5_6
Ex_9_L5_6
Ex_10_L2_4
Layer1
Layer2
Layer3
Layer4
Layer5
Layer6
WM
nan
Layers

B

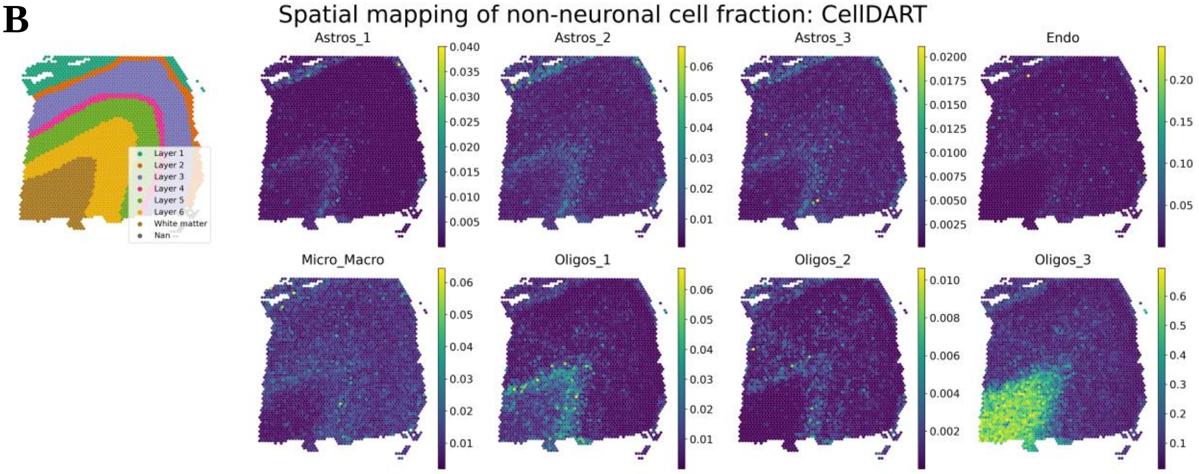

Spatial mapping of non-neuronal cell fraction: CellDART
Astros_1
Astros_2
Astros_3
Endo
Micro_Macro
Oligos_1
Oligos_2
Oligos_3
Layer 1
Layer 2
Layer 3
Layer 4
Layer 5
Layer 6
White matter
Nan

Figure S4: Discovery of spatial cell localization patterns by CellDART

(A) A stacked violin plot for the layer-specific excitatory neuron fraction predicted by CellDART. The distribution of a specific excitatory neuron fraction in the spots corresponding to one of the cortical layers (from layer 1 to 6), white matter (WM), or non-specified spots (nan) was presented in each row.

(B) Spatial mapping of glial cells [astrocytes (Astros) and oligodendrocytes (Oligos)], endothelial cells (Endo), and microglia or macrophages (Micro_Macro) fraction predicted by CellDART. The figure in the top left corner shows the layer annotation for each spatial spot. The layer consists of cortical layers 1 to 6 and white matter. ‘Nan’ represents the spot without the layer information. Colormaps present the maximum and minimum values for the corresponding cell fraction.

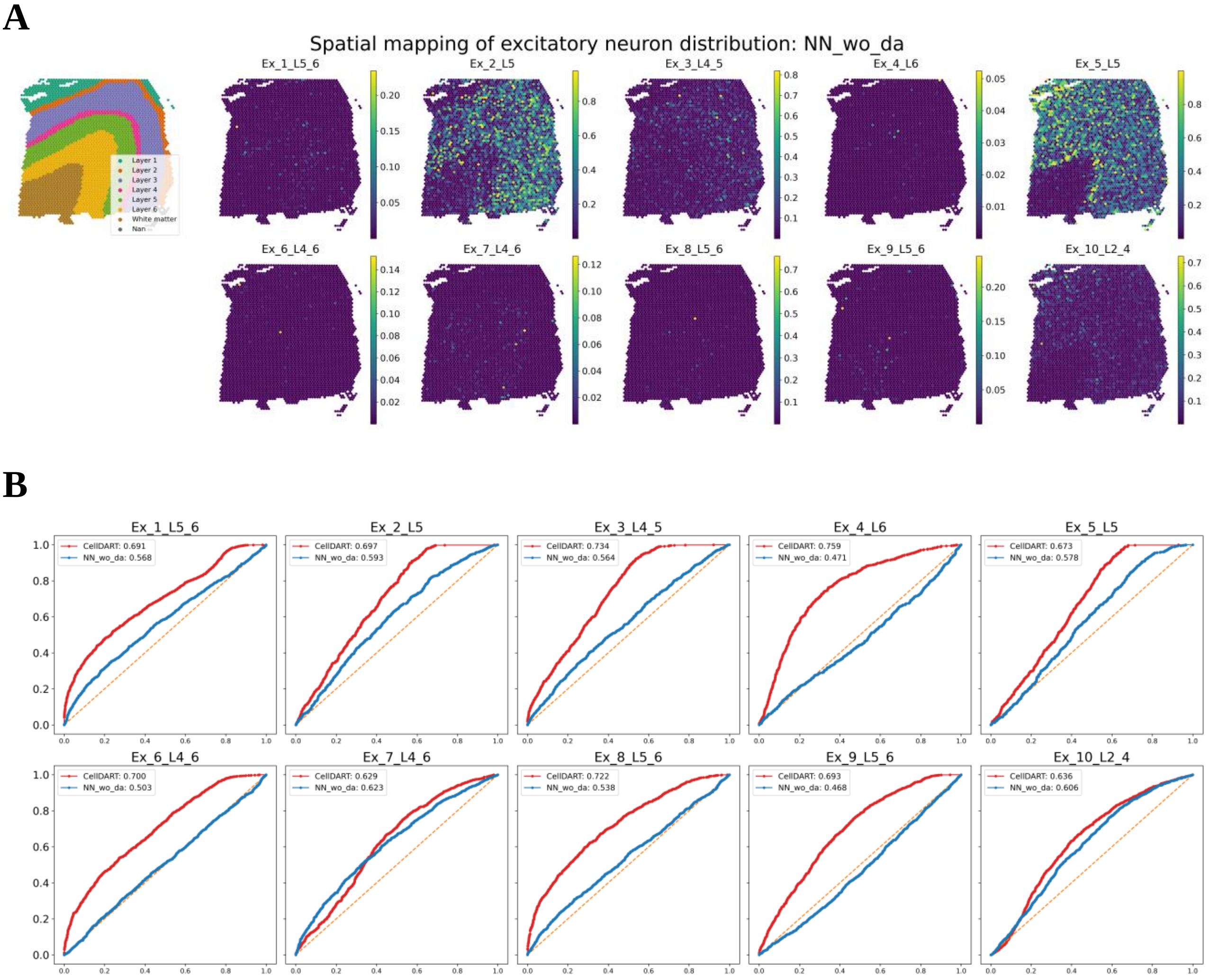
A
Spatial mapping of excitatory neuron distribution: NN_wo_da
Layer 1
Layer 2
Layer 3
Layer 4
Layer 5
Layer 6
White matter
Nan
Ex_1_L5_6
Ex_2_L5
Ex_3_L4_5
Ex_4_L6
Ex_5_L5
Ex_6_L4_6
Ex_7_L4_6
Ex_8_L5_6
Ex_9_L5_6
Ex_10_L2_4
B
Ex_1_L5_6
CellDART: 0.691
NN_wo_da: 0.568
Ex_2_L5
CellDART: 0.697
NN_wo_da: 0.593
Ex_3_L4_5
CellDART: 0.734
NN_wo_da: 0.564
Ex_4_L6
CellDART: 0.759
NN_wo_da: 0.471
Ex_5_L5
CellDART: 0.673
NN_wo_da: 0.578
Ex_6_L4_6
CellDART: 0.700
NN_wo_da: 0.503
Ex_7_L4_6
CellDART: 0.629
NN_wo_da: 0.623
Ex_8_L5_6
CellDART: 0.722
NN_wo_da: 0.538
Ex_9_L5_6
CellDART: 0.693
NN_wo_da: 0.468
Ex_10_L2_4
CellDART: 0.636
NN_wo_da: 0.606

Figure S5: Comparison of CellDART with a neural network without domain adaptation

(A) Spatial mapping of 10 layer-specific excitatory neuron fractions predicted by neural network model without domain adaptation (NN_wo_da). The figure in the top left corner shows the layer annotation for each spatial spot. The layer consists of cortical layers 1 to 6 and white matter. 'Nan' represents the spot without the layer information. Colormaps present the maximum and minimum values for the corresponding cell fraction.

(B) Receiver operating characteristic (ROC) analysis for predicting the layer-specific distribution of excitatory neurons. The CellDART (red) and NN_wo_da (blue) were compared using the area under the curve (AUC). The ROC curves for CellDART and NN_wo_da are color-coded, and AUC values are presented in the lower right corner of each plot.

A

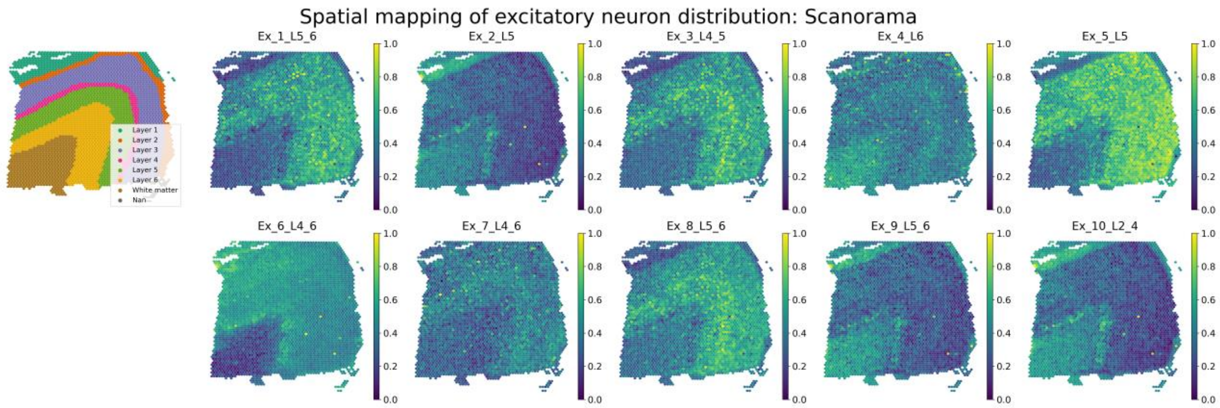
Spatial mapping of excitatory neuron distribution: Scanorama
Ex_1_L5_6
Ex_2_L5
Ex_3_L4_5
Ex_4_L6
Ex_5_L5
Ex_6_L4_6
Ex_7_L4_6
Ex_8_L5_6
Ex_9_L5_6
Ex_10_L2_4
Layer 1
Layer 2
Layer 3
Layer 4
Layer 5
Layer 6
White matter
Nan
0.0
0.2
0.4
0.6
0.8
1.0

B

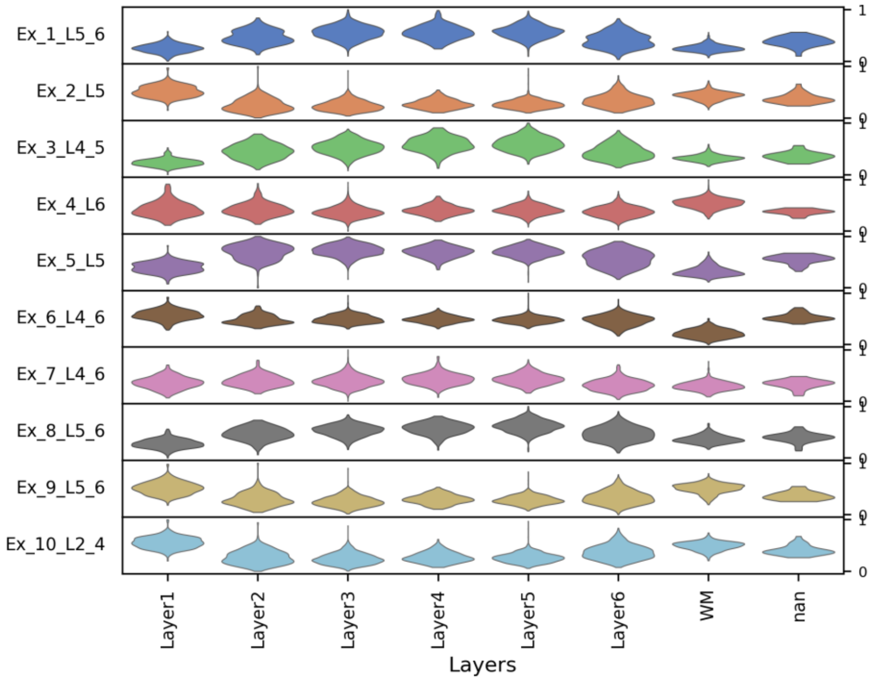
Ex_1_L5_6
Ex_2_L5
Ex_3_L4_5
Ex_4_L6
Ex_5_L5
Ex_6_L4_6
Ex_7_L4_6
Ex_8_L5_6
Ex_9_L5_6
Ex_10_L2_4
Layer1
Layer2
Layer3
Layer4
Layer5
Layer6
WM
nan
Layers
0
1

C

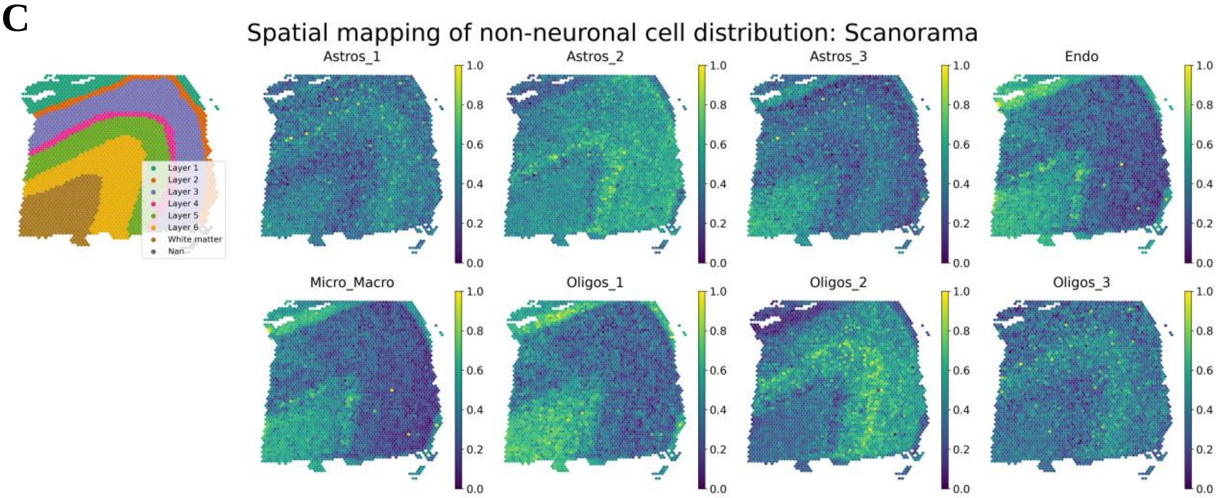
Spatial mapping of non-neuronal cell distribution: Scanorama
Astros_1
Astros_2
Astros_3
Endo
Micro_Macro
Oligos_1
Oligos_2
Oligos_3
Layer 1
Layer 2
Layer 3
Layer 4
Layer 5
Layer 6
White matter
Nan
0.0
0.2
0.4
0.6
0.8
1.0

Figure S6: Estimation of spatial cell proportion by Scanorama

(A) Spatial mapping of 10 layer-specific excitatory neuron fractions predicted by Scanorama. The figure in the top left corner shows the layer annotation for each spatial spot. The layer consists of cortical layers 1 to 6 and white matter. ‘Nan’ represents the spot without the layer information. Colormaps present the maximum and minimum values for the corresponding cell fraction.

(B) A stacked violin plot for the layer-specific excitatory neuron fraction predicted by Scanorama. The distribution of a specific excitatory neuron fraction in the spots corresponding to one of the cortical layers (from layer 1 to 6), white matter (WM), or non-specified spots (nan) was presented in each row.

(C) Spatial mapping of non-neuronal cells (Astros, Oligos, Endo, and Micro_Macro) fraction predicted by Scanorama. Colormaps present the maximum and minimum values for the corresponding cell fraction.

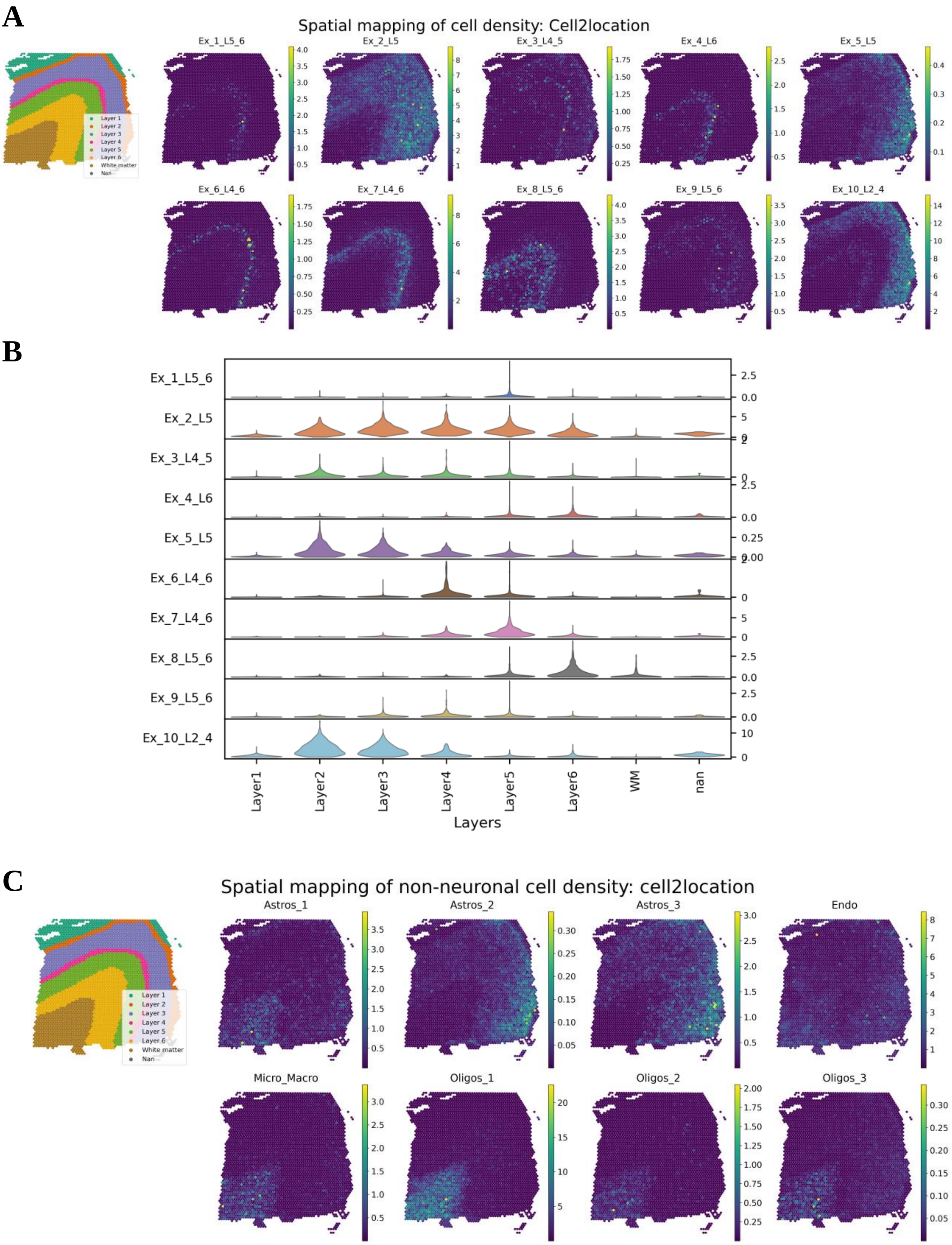
A
Spatial mapping of cell density: Cell2location
Ex_1_L5_6
Ex_2_L5
Ex_3_L4_5
Ex_4_L6
Ex_5_L5
Ex_6_L4_6
Ex_7_L4_6
Ex_8_L5_6
Ex_9_L5_6
Ex_10_L2_4
B
Ex_1_L5_6
Ex_2_L5
Ex_3_L4_5
Ex_4_L6
Ex_5_L5
Ex_6_L4_6
Ex_7_L4_6
Ex_8_L5_6
Ex_9_L5_6
Ex_10_L2_4
Layer1
Layer2
Layer3
Layer4
Layer5
Layer6
WM
nan
Layers
C
Spatial mapping of non-neuronal cell density: cell2location
Astros_1
Astros_2
Astros_3
Endo
Micro_Macro
Oligos_1
Oligos_2
Oligos_3

Figure S7: Prediction of spatial cell fraction by Cell2location

(A) Spatial mapping of 10 layer-specific excitatory neuron fractions predicted by Cell2location. The figure in the top left corner shows the layer annotation for each spatial spot. The layer consists of cortical layers 1 to 6 and white matter. ‘Nan’ represents the spot without the layer information. Colormaps present the maximum and minimum values for the corresponding cell fraction.

(B) A stacked violin plot for the layer-specific excitatory neuron fraction predicted by Cell2location. The distribution of a specific excitatory neuron fraction in the spots corresponding to one of the cortical layers (from layer 1 to 6), white matter (WM), or non-specified spots (nan) was presented in each row.

(C) Spatial mapping of non-neuronal cells (Astros, Oligos, Endo, and Micro_Macro) fraction predicted by Cell2location. Colormaps present the maximum and minimum values for the corresponding cell fraction.

A

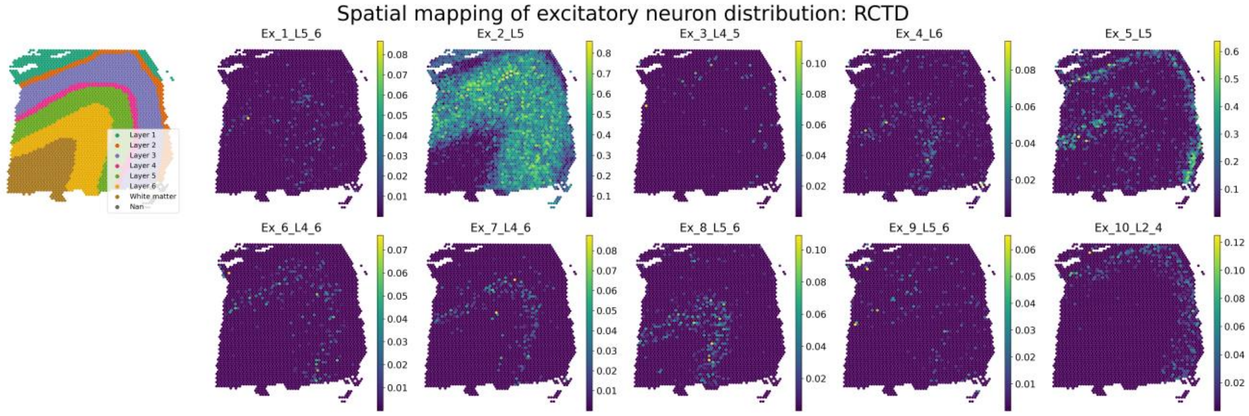
Spatial mapping of excitatory neuron distribution: RCTD
Ex_1_L5_6
Ex_2_L5
Ex_3_L4_5
Ex_4_L6
Ex_5_L5
Ex_6_L4_6
Ex_7_L4_6
Ex_8_L5_6
Ex_9_L5_6
Ex_10_L2_4
Layer 1
Layer 2
Layer 3
Layer 4
Layer 5
Layer 6
White matter
Nan

B

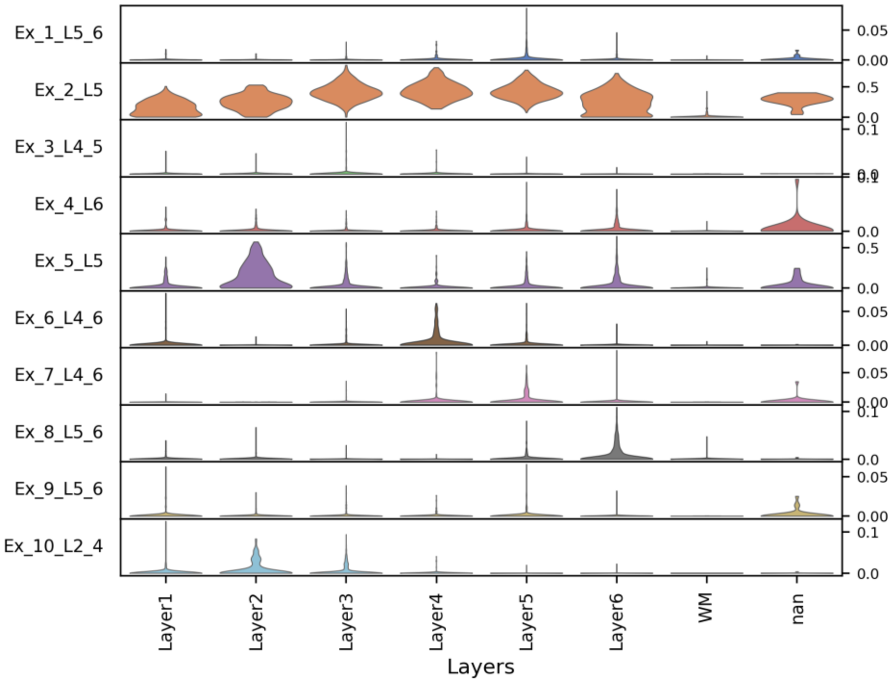
Ex_1_L5_6
Ex_2_L5
Ex_3_L4_5
Ex_4_L6
Ex_5_L5
Ex_6_L4_6
Ex_7_L4_6
Ex_8_L5_6
Ex_9_L5_6
Ex_10_L2_4
Layer1
Layer2
Layer3
Layer4
Layer5
Layer6
WM
nan
Layers

C

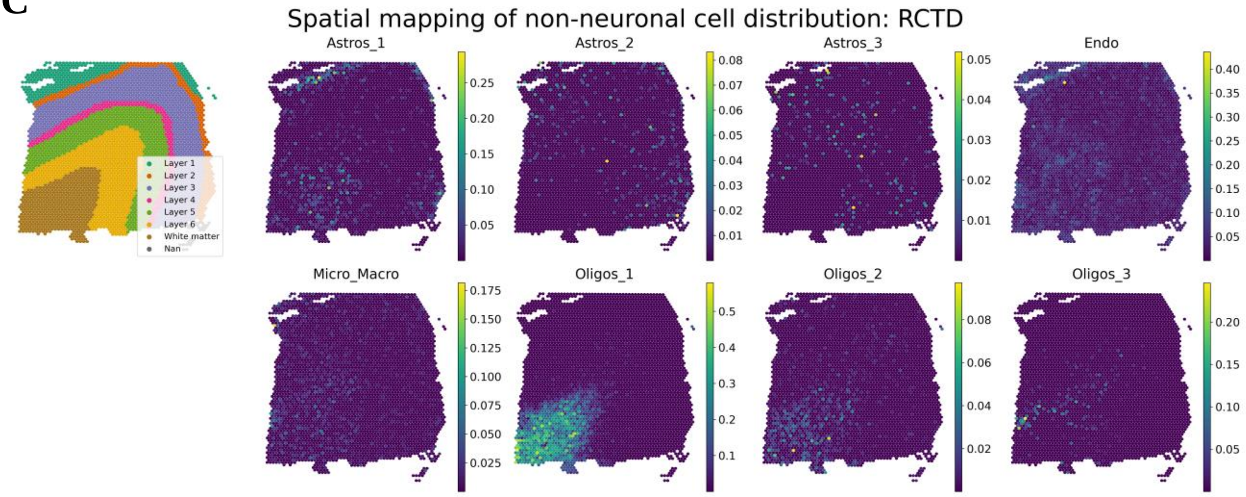
Spatial mapping of non-neuronal cell distribution: RCTD
Astros_1
Astros_2
Astros_3
Endo
Micro_Macro
Oligos_1
Oligos_2
Oligos_3
Layer 1
Layer 2
Layer 3
Layer 4
Layer 5
Layer 6
White matter
Nan

Figure S8: Estimation of spatial cell fraction by RCTD

(A) Spatial mapping of 10 layer-specific excitatory neuron fractions predicted by RCTD. The figure in the top left corner shows the layer annotation for each spatial spot. The layer consists of cortical layers 1 to 6 and white matter. 'Nan' represents the spot without the layer information. Colormaps present the maximum and minimum values for the corresponding cell fraction.

(B) A stacked violin plot for the layer-specific excitatory neuron fraction predicted by RCTD. The distribution of a specific excitatory neuron fraction in the spots corresponding to one of the cortical layers (from layer 1 to 6), white matter (WM), or non-specified spots (nan) was presented in each row.

(C) Spatial mapping of non-neuronal cells (Astros, Oligos, Endo, and Micro_Macro) fraction estimated by RCTD. Colormaps present the maximum and minimum values for the corresponding cell fraction.

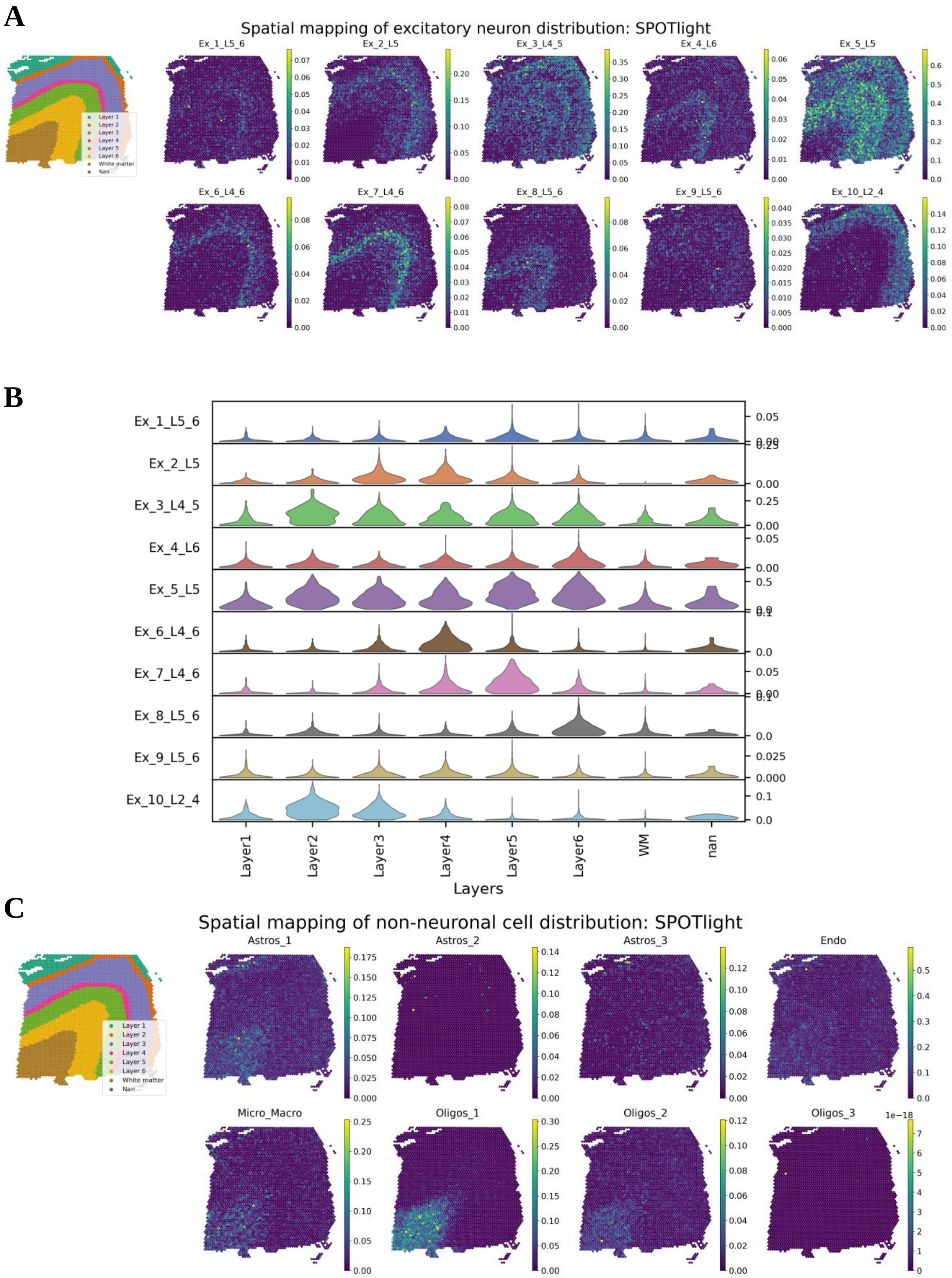
A
Spatial mapping of excitatory neuron distribution: SPOTlight
Ex_1_L5_6
Ex_2_L5
Ex_3_L4_5
Ex_4_L6
Ex_5_L5
Ex_6_L4_6
Ex_7_L4_6
Ex_8_L5_6
Ex_9_L5_6
Ex_10_L2_4
Layer 1
Layer 2
Layer 3
Layer 4
Layer 5
Layer 6
White matter
Nan
B
Ex_1_L5_6
Ex_2_L5
Ex_3_L4_5
Ex_4_L6
Ex_5_L5
Ex_6_L4_6
Ex_7_L4_6
Ex_8_L5_6
Ex_9_L5_6
Ex_10_L2_4
Layer1
Layer2
Layer3
Layer4
Layer5
Layer6
WM
nan
Layers
C
Spatial mapping of non-neuronal cell distribution: SPOTlight
Astros_1
Astros_2
Astros_3
Endo
Micro_Macro
Oligos_1
Oligos_2
Oligos_3
1e−18

Figure S9: Prediction of spatial cell proportion by SPOTlight

(A) Spatial mapping of 10 layer-specific excitatory neuron fractions predicted by SPOTlight. The figure in the top left corner shows the layer annotation for each spatial spot. The layer consists of cortical layers 1 to 6 and white matter. 'Nan' represents the spot without the layer information. Colormaps present the maximum and minimum values for the corresponding cell fraction.

(B) A stacked violin plot for the layer-specific excitatory neuron fraction predicted by SPOTlight. The distribution of a specific excitatory neuron fraction in the spots corresponding to one of the cortical layers (from layer 1 to 6), white matter (WM), or non-specified spots (nan) was presented in each row.

(C) Spatial mapping of non-neuronal cells (Astros, Oligos, Endo, and Micro_Macro) fraction estimated by SPOTlight. Colormaps present the maximum and minimum values for the corresponding cell fraction.

A

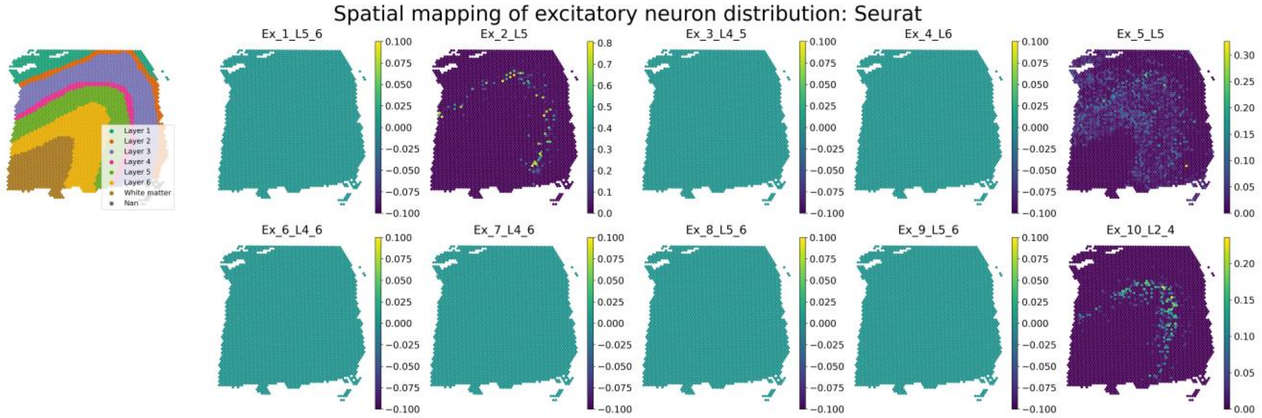
Spatial mapping of excitatory neuron distribution: Seurat
Ex_1_L5_6
Ex_2_L5
Ex_3_L4_5
Ex_4_L6
Ex_5_L5
Ex_6_L4_6
Ex_7_L4_6
Ex_8_L5_6
Ex_9_L5_6
Ex_10_L2_4

B

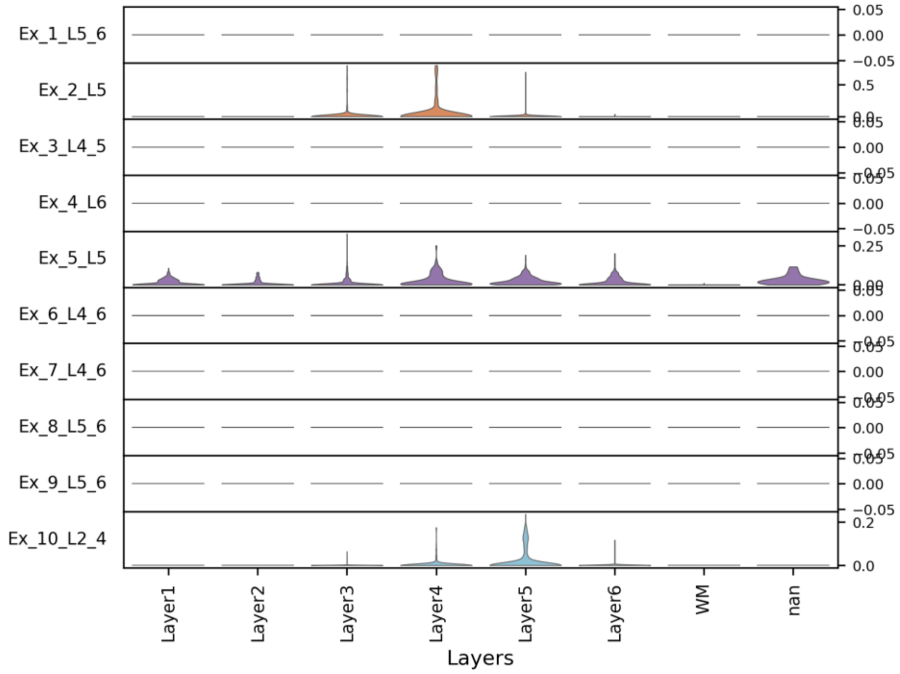
Ex_1_L5_6
Ex_2_L5
Ex_3_L4_5
Ex_4_L6
Ex_5_L5
Ex_6_L4_6
Ex_7_L4_6
Ex_8_L5_6
Ex_9_L5_6
Ex_10_L2_4
Layer1
Layer2
Layer3
Layer4
Layer5
Layer6
WM
nan
Layers

C

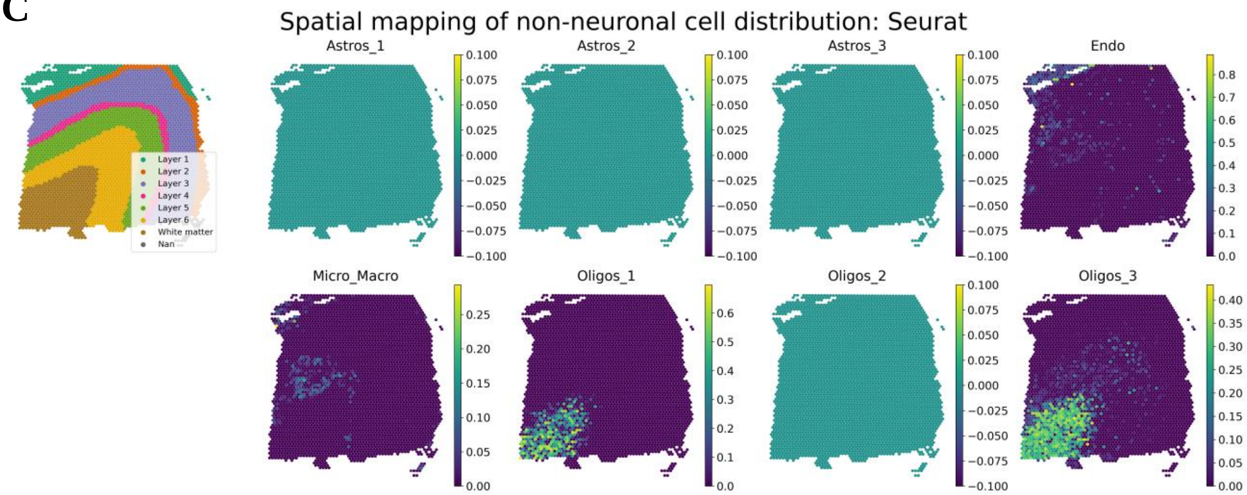
Spatial mapping of non-neuronal cell distribution: Seurat
Astros_1
Astros_2
Astros_3
Endo
Micro_Macro
Oligos_1
Oligos_2
Oligos_3

Figure S10: Prediction of spatial cell proportion by Seurat

(A) Spatial mapping of 10 layer-specific excitatory neuron fractions predicted by Seurat. The figure in the top left corner shows the layer annotation for each spatial spot. The layer consists of cortical layers 1 to 6 and white matter. 'Nan' represents the spot without the layer information. Colormaps present the maximum and minimum values for the corresponding cell fraction. In some excitatory neuron types, Seurat predicted the cell fraction to be 0 in all the spots.

(B) A stacked violin plot for the layer-specific excitatory neuron fraction predicted by Seurat. The distribution of a specific excitatory neuron fraction in the spots corresponding to one of the cortical layers (from layer 1 to 6), white matter (WM), or non-specified spots (nan) was presented in each row.

(C) Spatial mapping of non-neuronal cells (Astros, Oligos, Endo, and Micro_Macro) fraction estimated by Seurat. In some cell types, Seurat predicted the cell fraction to be 0 in all the spots. Colormaps present the maximum and minimum values for the corresponding cell fraction.

A

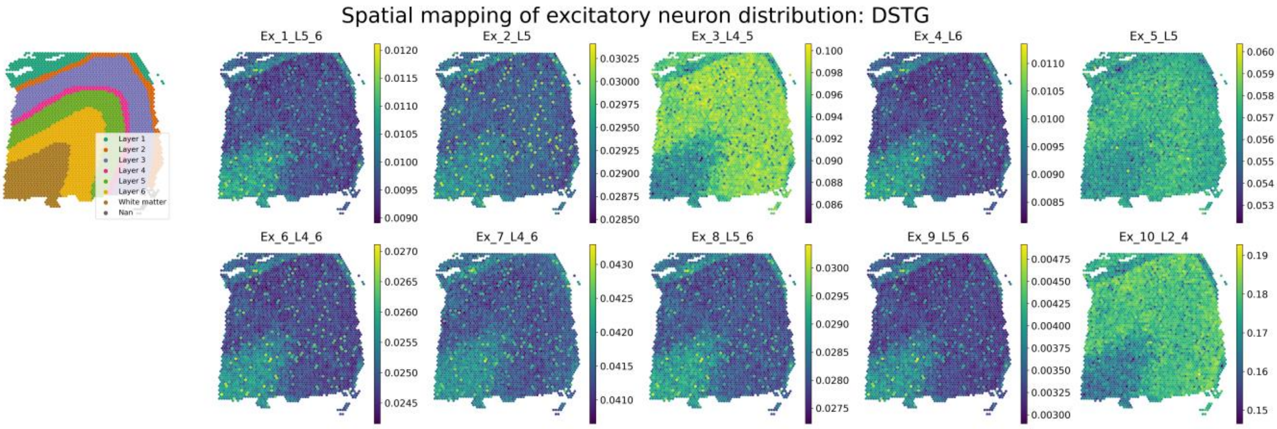
Spatial mapping of excitatory neuron distribution: DSTG
Ex_1_L5_6
Ex_2_L5
Ex_3_L4_5
Ex_4_L6
Ex_5_L5
Ex_6_L4_6
Ex_7_L4_6
Ex_8_L5_6
Ex_9_L5_6
Ex_10_L2_4

B

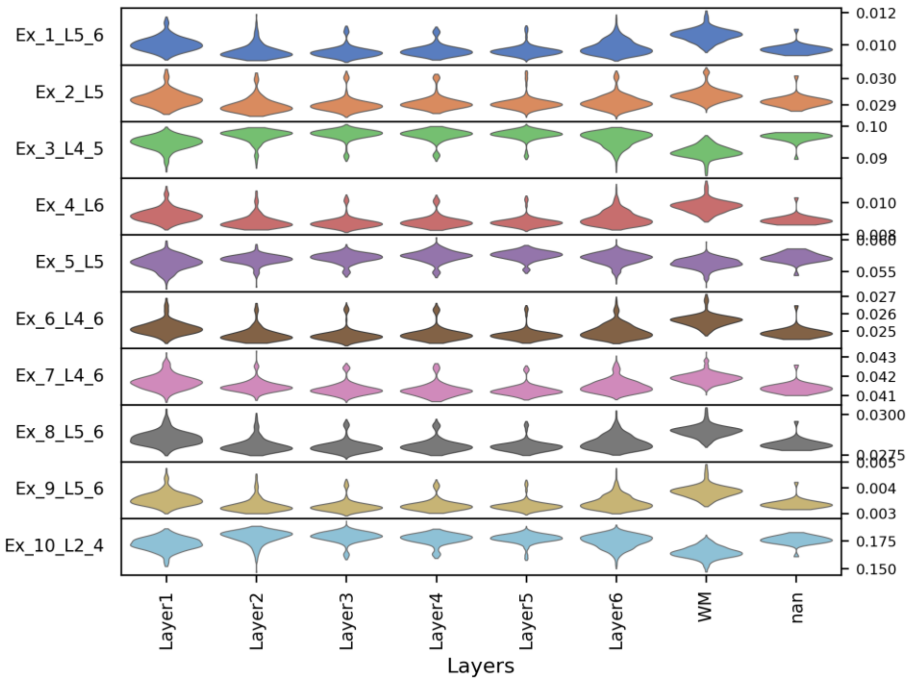
Ex_1_L5_6
Ex_2_L5
Ex_3_L4_5
Ex_4_L6
Ex_5_L5
Ex_6_L4_6
Ex_7_L4_6
Ex_8_L5_6
Ex_9_L5_6
Ex_10_L2_4
Layer1
Layer2
Layer3
Layer4
Layer5
Layer6
WM
nan
Layers

C

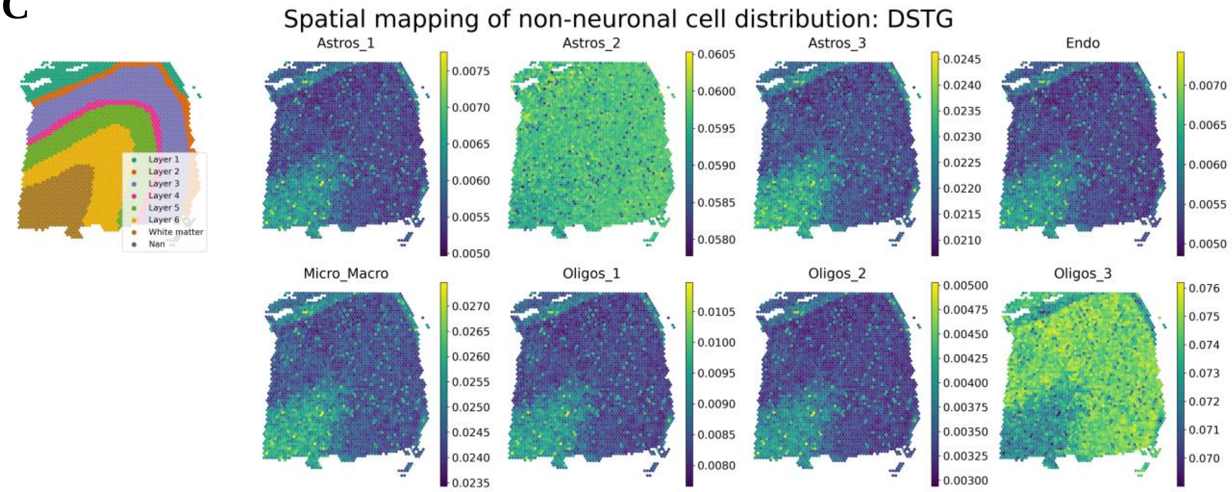
Spatial mapping of non-neuronal cell distribution: DSTG
Astros_1
Astros_2
Astros_3
Endo
Micro_Macro
Oligos_1
Oligos_2
Oligos_3

Figure S11: Prediction of spatial cell composition by DSTG

(A) Spatial mapping of 10 layer-specific excitatory neuron fractions predicted by DSTG. The figure in the top left corner shows the layer annotation for each spatial spot. The layer consists of cortical layers 1 to 6 and white matter. 'Nan' represents the spot without the layer information. Colormaps present the maximum and minimum values for the corresponding cell fraction.

(B) A stacked violin plot for the layer-specific excitatory neuron fraction predicted by DSTG. The distribution of a specific excitatory neuron fraction in the spots corresponding to one of the cortical layers (from layer 1 to 6), white matter (WM), or non-specified spots (nan) was presented in each row.

(C) Spatial mapping of non-neuronal cells (Astros, Oligos, Endo, and Micro_Macro) fraction estimated by DSTG. Colormaps present the maximum and minimum values for the corresponding cell fraction.

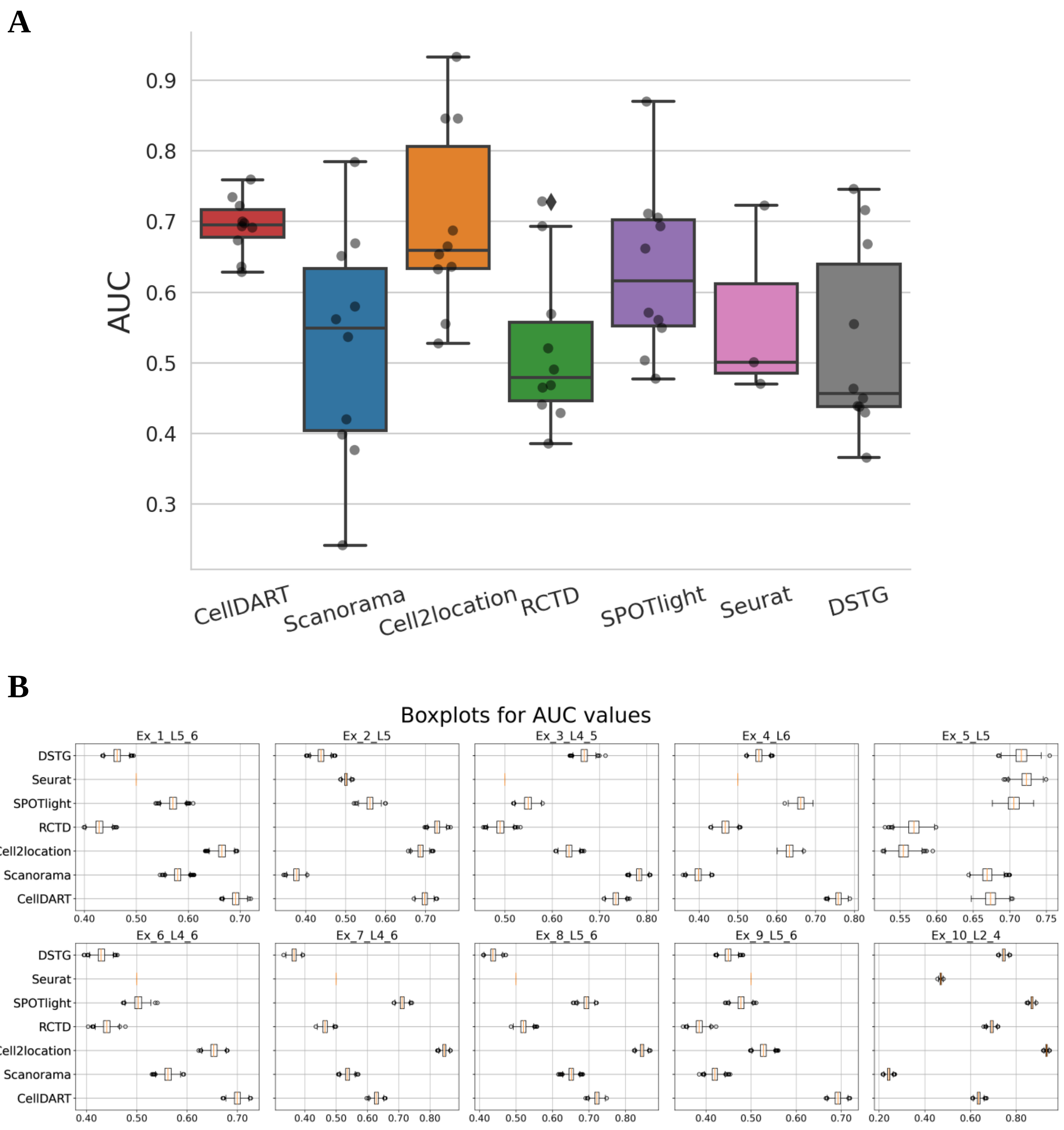
A
AUC
0.9
0.8
0.7
0.6
0.5
0.4
0.3
CellDART
Scanorama
Cell2location
RCTD
SPOTlight
Seurat
DSTG
B
Boxplots for AUC values
Ex_1_L5_6
Ex_2_L5
Ex_3_L4_5
Ex_4_L6
Ex_5_L5
Ex_6_L4_6
Ex_7_L4_6
Ex_8_L5_6
Ex_9_L5_6
Ex_10_L2_4
DSTG
Seurat
SPOTlight
RCTD
Cell2location
Scanorama
CellDART

Figure S12: Comparison of AUC values for predicting the layer-specific distribution of excitatory neurons in the DLPFC 151673 dataset.

(A) The AUC values for the 10 excitatory neurons were collected and their distribution across seven computational tools was visualized with a boxplot. In the case of Seurat, the spatial cell fraction was predicted to be all 0 in a majority of cases (7/10) and they were not visualized. Grey points in the boxplot represent the individual AUC values for each excitatory neuron subtype.

(B) The cell fraction values and layer labels of spatial spots for 10 excitatory neurons were bootstrapped and the 1,000 null distribution for the AUC values were obtained. The results derived from seven different tools, CellDART, Scanorma, Cell2location, RCTD, SPOTlight, Seurat, and DSTG were visualized with boxplots. In the case of Seurat, it failed to predict cell composition in a majority of cases and they were visualized as having an AUC of 0.5.

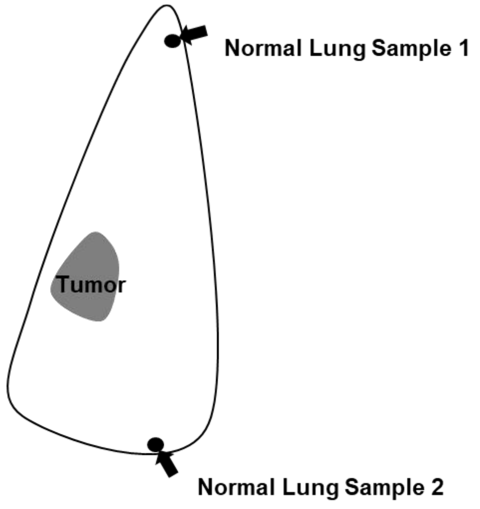

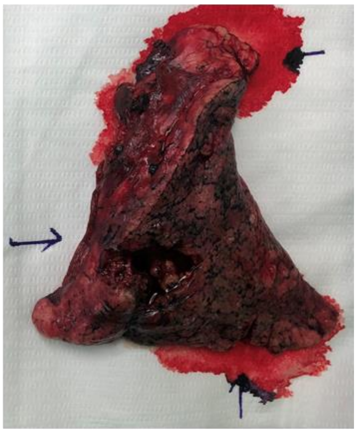

Figure S13: Gross specimen of human lung tissue 1 and 2 data

A

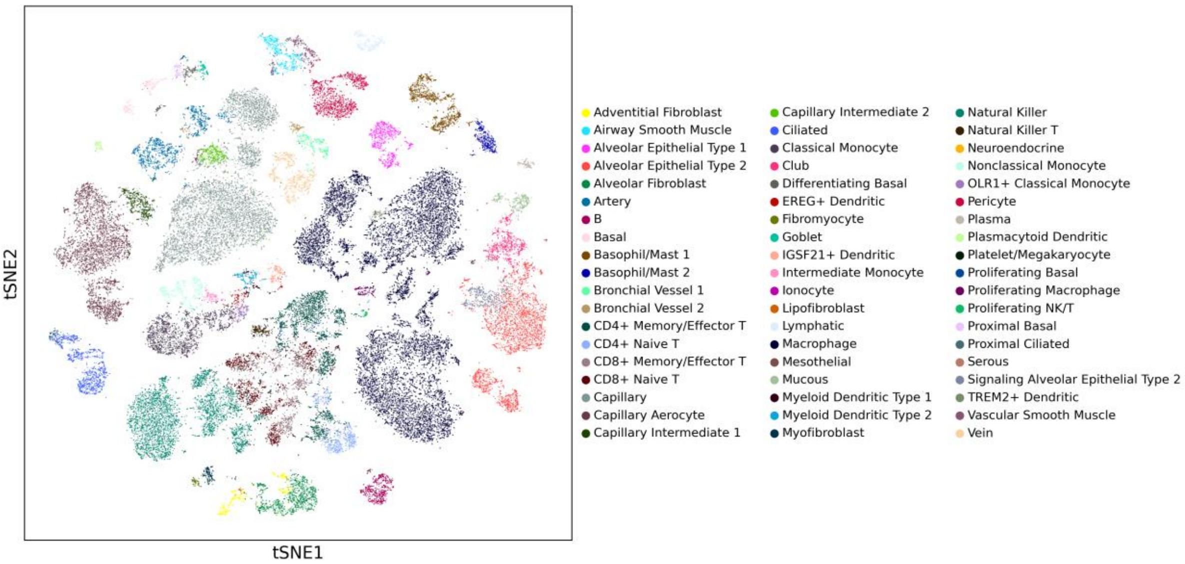

B

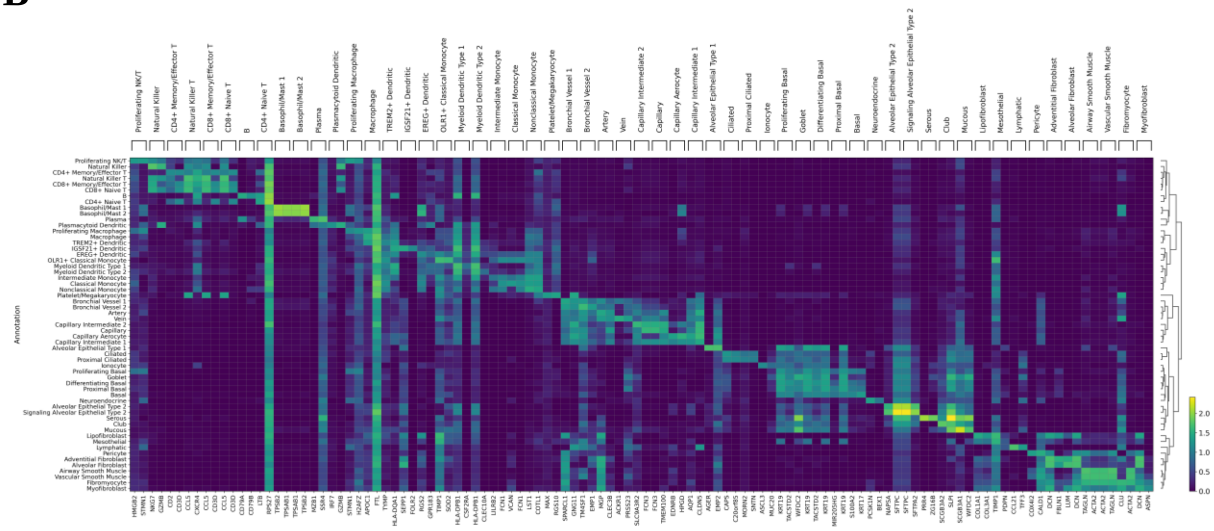

Figure S14: Preprocessing human lung single-cell data

(A) t-SNE map for gene expression in lung single-cell data. The identity of the 57 clusters is color-coded and presented on the right side of the plot.

(B) A heatmap for average log-normalized gene expression in the top 2 marker genes of each cell cluster. Hierarchical clustering was performed for the 57 cell types based on the expression profiles of the presented genes.

A

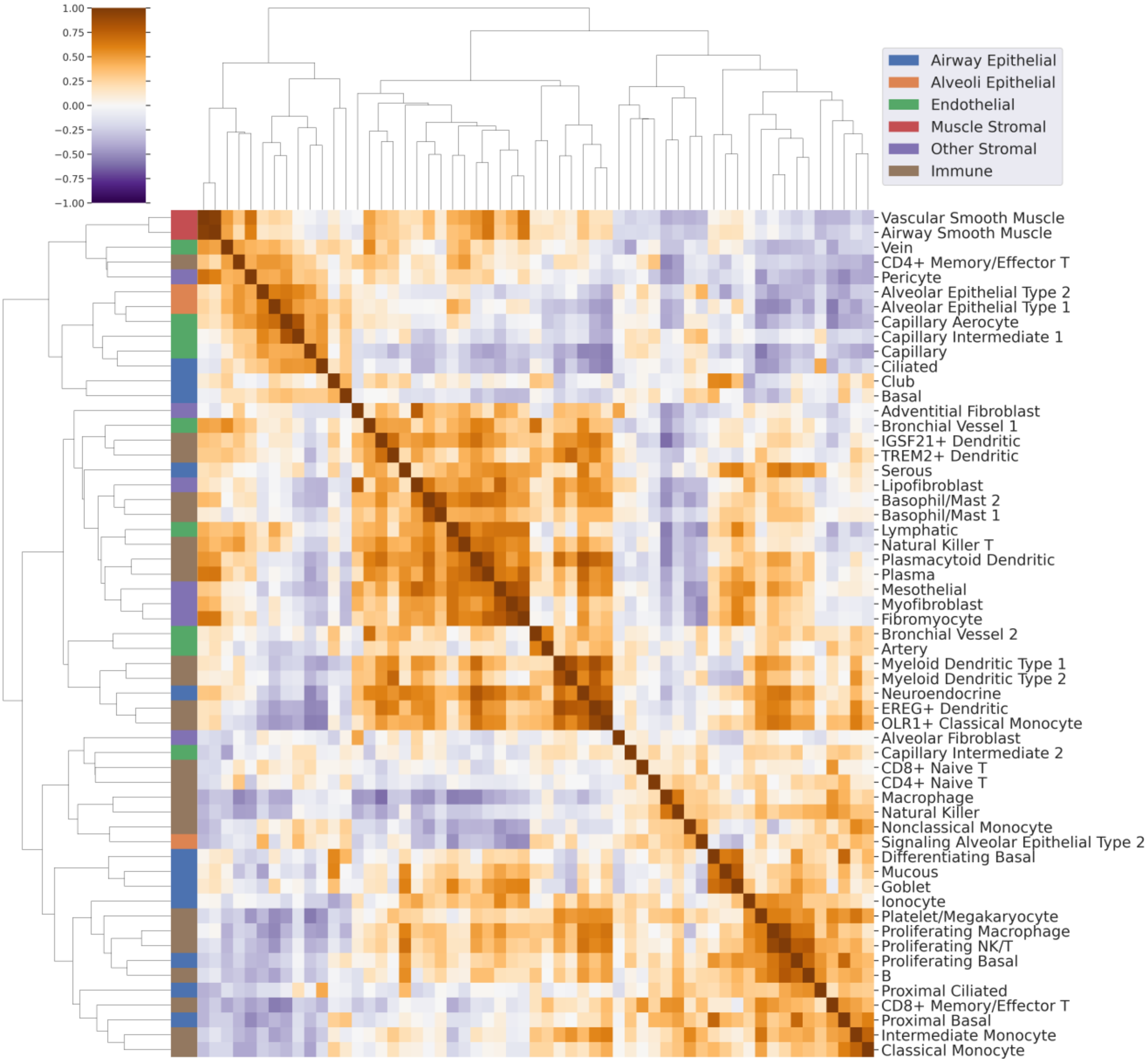
1.00
0.75
0.50
0.25
0.00
−0.25
−0.50
−0.75
−1.00
Airway Epithelial
Alveoli Epithelial
Endothelial
Muscle Stromal
Other Stromal
Immune
Vascular Smooth Muscle
Airway Smooth Muscle
Vein
CD4+ Memory/Effector T
Pericyte
Alveolar Epithelial Type 2
Alveolar Epithelial Type 1
Capillary Aerocyte
Capillary Intermediate 1
Capillary
Ciliated
Club
Basal
Adventitial Fibroblast
Bronchial Vessel 1
IGSF21+ Dendritic
TREM2+ Dendritic
Serous
Lipofibroblast
Basophil/Mast 2
Basophil/Mast 1
Lymphatic
Natural Killer T
Plasmacytoid Dendritic
Plasma
Mesothelial
Myofibroblast
Fibromyocyte
Bronchial Vessel 2
Artery
Myeloid Dendritic Type 1
Myeloid Dendritic Type 2
Neuroendocrine
EREG+ Dendritic
OLR1+ Classical Monocyte
Alveolar Fibroblast
Capillary Intermediate 2
CD8+ Naive T
CD4+ Naive T
Macrophage
Natural Killer
Nonclassical Monocyte
Signaling Alveolar Epithelial Type 2
Differentiating Basal
Mucous
Goblet
Ionocyte
Platelet/Megakaryocyte
Proliferating Macrophage
Proliferating NK/T
Proliferating Basal
B
Proximal Ciliated
CD8+ Memory/Effector T
Proximal Basal
Intermediate Monocyte
Classical Monocyte

B

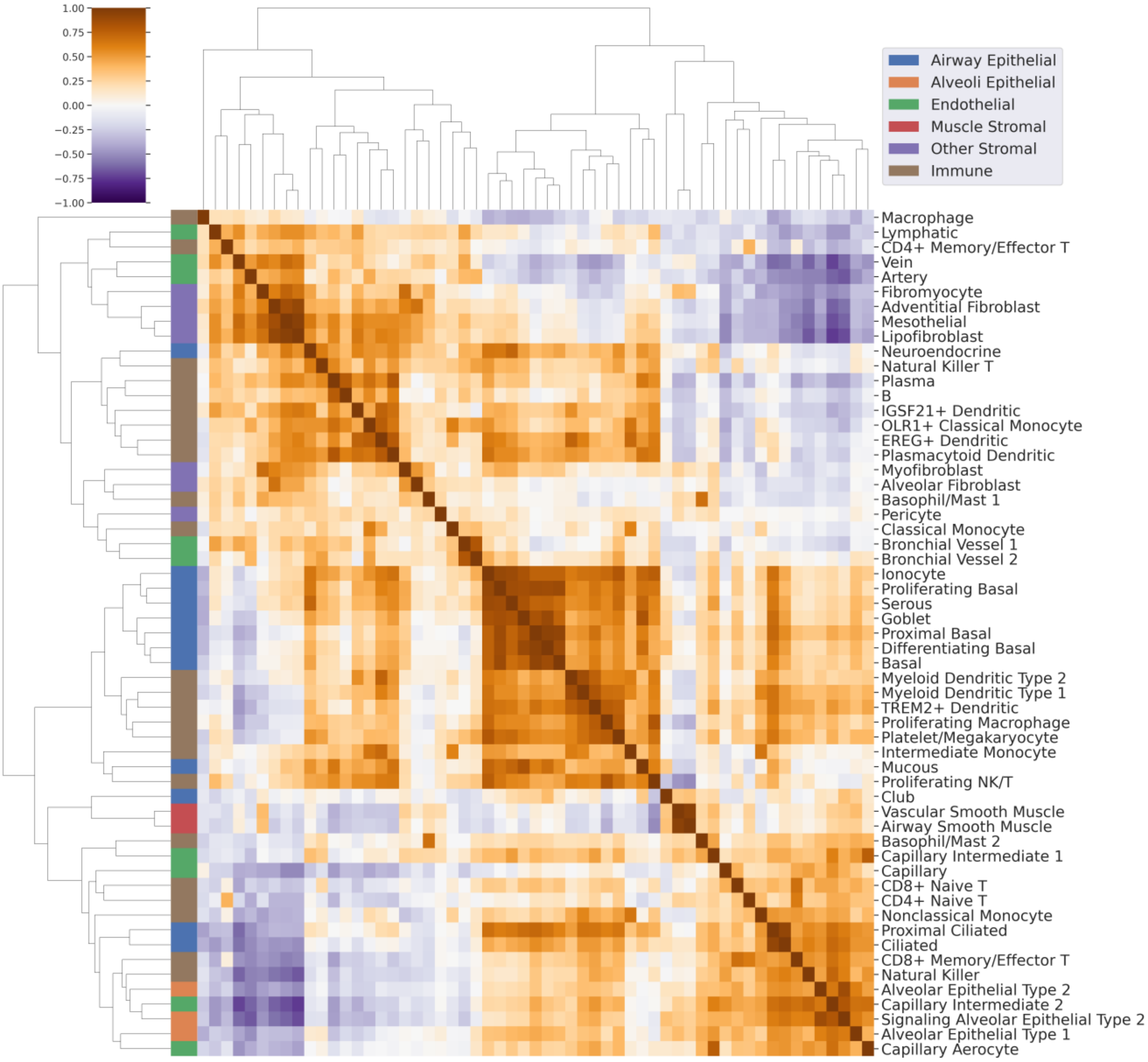
1.00
0.75
0.50
0.25
0.00
−0.25
−0.50
−0.75
−1.00
Airway Epithelial
Alveoli Epithelial
Endothelial
Muscle Stromal
Other Stromal
Immune
Macrophage
Lymphatic
CD4+ Memory/Effector T
Vein
Artery
Fibromyocyte
Adventitial Fibroblast
Mesothelial
Lipofibroblast
Neuroendocrine
Natural Killer T
Plasma
B
IGSF21+ Dendritic
OLR1+ Classical Monocyte
EREG+ Dendritic
Plasmacytoid Dendritic
Myofibroblast
Alveolar Fibroblast
Basophil/Mast 1
Pericyte
Classical Monocyte
Bronchial Vessel 1
Bronchial Vessel 2
Ionocyte
Proliferating Basal
Serous
Goblet
Proximal Basal
Differentiating Basal
Basal
Myeloid Dendritic Type 2
Myeloid Dendritic Type 1
TREM2+ Dendritic
Proliferating Macrophage
Platelet/Megakaryocyte
Intermediate Monocyte
Mucous
Proliferating NK/T
Club
Vascular Smooth Muscle
Airway Smooth Muscle
Basophil/Mast 2
Capillary Intermediate 1
Capillary
CD8+ Naive T
CD4+ Naive T
Nonclassical Monocyte
Proximal Ciliated
Ciliated
CD8+ Memory/Effector T
Natural Killer
Alveolar Epithelial Type 2
Capillary Intermediate 2
Signaling Alveolar Epithelial Type 2
Alveolar Epithelial Type 1
Capillary Aerocyte

Figure S15: Spatial correlation maps between various cell types in normal lung tissue

Heatmaps for the Spearman's correlation coefficient between spatial cell fraction of two different cell types in (A) lung 1 and (B) 2 tissues. The cell types were classified into 6 categories ('alveolar epithelial', 'alveoli epithelial', 'endothelial', 'muscle stromal', 'other stromal', and 'immune') based on the original paper of the human lung cell atlas (32) and color-coded on the left side of the heatmaps. Additionally, hierarchical clustering was performed based on correlation values between spatial cell fractions to visualize the spatial similarity between the cell types.
